# Unveiling potential virulence determinants in *Vibrio* isolates from *Anadara tuberculosa* through whole genome analyses

**DOI:** 10.1128/spectrum.02928-23

**Published:** 2024-01-08

**Authors:** Mariana Restrepo-Benavides, Daniela Lozano-Arce, Laura Natalia Gonzalez-Garcia, Felipe Báez-Aguirre, Gabriela Ariza-Aranguren, Daniel Faccini, María Mercedes Zambrano, Pedro Jiménez, Ana Fernández-Bravo, Silvia Restrepo, Marcela Guevara-Suarez

**Affiliations:** 1 Applied Genomics Research Group, Vicerrectoría de Investigación y Creación, Universidad de los Andes, Bogotá, Colombia; 2 Unit of Microbiology, Department of Basic Health Sciences, Faculty of Medicine and Health Sciences, IISPV, University Rovira i Virgili, Reus, Spain; 3 Systems and Computing Engineering Department, Universidad de Los Andes, Bogotá, Colombia; 4 UMR DIADE, Institut de Recherche pour le Développement, Université de Montpellier, Montpellier, France; 5 Molecular Genetics, Corporación CorpoGen - Research Center, Bogotá, Colombia; 6 Laboratorio de Fitopatología, Facultad de Ciencias Básicas y Aplicadas, Universidad Militar Nueva Granada, Cajicá, Colombia; 7 Departamento de Ingeniería Química y de Alimentos, Laboratorio de Micología y Fitopatología, Universidad de los Andes, Bogotá, Colombia; Universidad Andres Bello, Santiago, Chile

**Keywords:** bivalve, Oxford Nanopore, piangua, GridION, secretion systems, multidrug resistance

## Abstract

The genus *Vibrio* includes pathogenic bacteria able to cause disease in humans and aquatic organisms, leading to disease outbreaks and significant economic losses in the fishery industry. Despite much work on *Vibrio* in several marine organisms, no specific studies have been conducted on *Anadara tuberculosa*. This is a commercially important bivalve species, known as “piangua hembra,” along Colombia’s Pacific coast. Therefore, this study aimed to identify and characterize the genomes of *Vibrio* isolates obtained from *A. tuberculosa*. Bacterial isolates were obtained from 14 *A. tuberculosa* specimens collected from two locations along the Colombian Pacific coast, of which 17 strains were identified as *Vibrio*: *V. parahaemolyticus* (*n* = 12), *V. alginolyticus* (*n* = 3), *V. fluvialis* (*n* = 1), and *V. natriegens* (*n* = 1). Whole genome sequence of these isolates was done using Oxford Nanopore Technologies (ONT). The analysis revealed the presence of genes conferring resistance to β-lactams, tetracyclines, chloramphenicol, and macrolides, indicating potential resistance to these antimicrobial agents. Genes associated with virulence were also found, suggesting the potential pathogenicity of these *Vibrio* isolates, as well as genes for Type III Secretion Systems (T3SS) and Type VI Secretion Systems (T6SS), which play crucial roles in delivering virulence factors and in interbacterial competition. This study represents the first genomic analysis of bacteria within *A. tuberculosa*, shedding light on *Vibrio* genetic factors and contributing to a comprehensive understanding of the pathogenic potential of these *Vibrio* isolates.

**IMPORTANCE:**

This study presents the first comprehensive report on the whole genome analysis of *Vibrio* isolates obtained from *Anadara tuberculosa*, a bivalve species of great significance for social and economic matters on the Pacific coast of Colombia. Research findings have significant implications for the field, as they provide crucial information on the genetic factors and possible pathogenicity of *Vibrio* isolates associated with *A. tuberculosa*. The identification of antimicrobial resistance genes and virulence factors within these isolates emphasizes the potential risks they pose to both human and animal health. Furthermore, the presence of genes associated with Type III and Type VI Secretion Systems suggests their critical role in virulence and interbacterial competition. Understanding the genetic factors that contribute to *Vibrio* bacterial virulence and survival strategies within their ecological niche is of utmost importance for the effective prevention and management of diseases in aquaculture practices.

## INTRODUCTION

*Vibrio* is a genus of Gram-negative, halophilic, and facultatively anaerobic bacteria commonly found in aquatic environments, including marine, estuarine, and freshwater habitats, and associated with different organisms such as mollusks and bivalves (1, 2). This bacterial genus plays a relevant role in the marine organic carbon cycle, which is crucial for the environment and benefits marine life (3). However, some *Vibrio* species are pathogenic to humans and aquatic animals causing significant economic losses to the aquaculture industry (4–6). Reported symptoms in humans infected with *Vibrio* bacteria range from diarrheal disease to deadly septicemia or cholera (7), whereas in aquatic animals, the symptoms range from ulcers to necrotic disease (8, 9). The incidence of vibriosis is increasing worldwide, and the increase in sea temperatures due to global warming favors the spread of pathogenic *Vibrio* species (2, 10, 11).

Among the more than 100 *Vibrio* species identified, *Vibrio cholerae*, *Vibrio parahaemolyticus*, and *Vibrio vulnificus* are the most important human pathogens (2). In contrast, known marine animal pathogens are *Vibrio alginolyticus*, *Vibrio anguillarum*, *Vibrio harveyi*, and *Vibrio fluvialis* (2, 4, 11). Pathogenicity of these species is determined by multiple virulence factors, which include genes for adhesion factors, biofilm formation, production of exotoxins, capsules and polysaccharides, quorum sensing molecules, iron acquisition systems, and Type III and Type VI Secretion Systems (T3SS and T6SS) (12, 13, 14). Cytotoxins produced by *Vibrio*, such as cholera toxin (CTX) and hemolysin, can cause tissue damage and cell death (15–17). Overall, the pathogenesis of *Vibrio* infections is complex and multifactorial, requiring the coordinated action of multiple virulence factors such as toxins, T3SS, and T6SS, and exotoxins such as hemolysins, among others (13).

Raw or partially cooked shellfish, such as bivalves, have been long recognized as a primary vehicle for *Vibrio* foodborne disease (18). However, little is known regarding most bivalve species from developing countries or their associated bacterial communities. The “piangua hembra,” *Anadara tuberculosa* Sowerby (1833), is the most abundant of the economically important bivalves along the Pacific coast between Perú and México (19, 20). It is associated with mangrove ecosystems, specifically with *Rhizophora mangle* roots, and it is harvested manually by local communities for subsistence and market trade (20, 21).

While recent studies on *A. tuberculosa* have focused on coliform concentration (22) and identifying probiotic bacteria for shrimp-farming fields (23), there remains a significant gap in research addressing microbial communities associated with this bivalve. Importantly, none of these studies have employed genomic sequencing or molecular techniques to comprehensively characterize these microorganisms (22, 24). This research gap is of paramount significance, given that *Vibrio* infections can cause various illnesses in humans and ulcers and necrotic diseases in aquatic animals (5, 8). The global occurrence of vibriosis is increasing due to warming seas, underscoring the need to study bivalve-related health risks (2).

Genomics constitutes a valuable tool for identifying and contrasting *Vibrio* species and their virulence genes. Therefore, this study aimed to identify and characterize *Vibrio* isolates associated with *A. tuberculosa* from the Colombian Pacific coast, using complete genome sequencing by ONT to assess their potential as pathogens and investigate possible antibiotic resistance.

## MATERIALS AND METHODS

### Isolation of cultivable bacteria

Specimens of *A. tuberculosa* were collected from two regions along the Colombian Pacific coast: (i) Buenaventura, Valle del Cauca and (ii) Iscuandé, Nariño. In Buenaventura, collections were made at three different sites, whereas in Iscuandé, specimens were obtained from four distinct locations, resulting in a total of seven collection sites (see Table S1 for details). At each site, two specimens were collected, resulting in a total of 14 specimens. Each oyster underwent an external rinsing with sterile water, followed by meticulous removal of the shell biofilm. The bivalves were then opened using a sterile knife. Subsequently, the entire fleshy body of each specimen was homogenized and inoculated on nutrient agar medium (Oxoid, UK) that was prepared using seawater collected from the sampling sites. This seawater, with an approximate salinity of 14 ppt, had been previously filtered to eliminate sediment particles and sterilized. After 24 hours of incubation at 25°C, different bacterial morphotypes were isolated on the same agar medium and preserved in nutrient broth medium (Oxoid, UK) containing 15% glycerol and stored at −80°C. The preserved bacteria were cultured on nutrient agar medium (Oxoid, UK) supplemented with 3% sea salt and incubated at 25°C for subsequent assays.

### Species identification and phylogenetic analysis of *Vibrio* isolates

To identify the bacterial strains, a colony PCR was conducted for each isolate, amplifying the 16S rRNA gene by PCR using 27F (5′-AGA GTT TGA TCC TGG CTC AG-3′) and 1492R (5′-GGT TAC CTT GTT ACG ACT T-3′) primers. Following the manufacturer’s instructions, PCR amplification was performed using GoTaq DNA Polymerase (Promega, USA) and the following PCR conditions: initial denaturation at 95°C for 5 minutes, 30 cycles of denaturation at 94°C for 30 seconds, annealing at 54°C for 30 seconds, and extension at 72°C for 60 seconds, then a final extension of 72°C for 10 minutes. PCR products were sequenced using Sanger technologies sequencing with an Applied Biosystems 3500XL Genetic Analyzer (Thermo Fisher Scientific, USA). Sequence assembly and editing were performed using Geneious Prime V 2023.0.4 (25).

The phylogenetic tree was constructed using the neighbor-joining method with default parameters and 1,000 bootstrap replications, utilizing the MEGA v.7 software (26), based on the 16S rRNA gene sequences of our isolates (Table S2) and reference strains (Table S3).

### Genomic DNA extraction and sequencing

*Vibrio* isolates were inoculated in 5 mL nutrient broth supplemented with 3% sea salt and incubated at 120 rpm in an orbital shaker at 25°C for 24 hours. Afterward, 4 mL of the culture was centrifuged at 13,000 rpm for 1 minute to obtain a pellet used for DNA extraction. Genomic DNA was extracted from each pellet using the DNeasy Blood and Tissue Kit (Qiagen, UK), following the instructions for the pretreatment of Gram-negative bacteria and the “Purification of Total DNA from Animal Tissues” protocol (Qiagen, UK). Finally, each sample was eluted with 50 µL of DNAse-free water. The elution process was repeated using the last elution to increase the final concentration of DNA. The DNA concentration and integrity were assessed using the Qubit Fluorometer 4.0 (Thermo Fisher Scientific, USA) and 1% agarose gel electrophoresis.

Genomic DNA sequencing was performed in the Sequencing Core Facility - GenCore (Universidad de Los Andes, Bogotá, Colombia) using the Oxford Nanopore Technologies (ONT) platform. To include multiple strains per single flow cell, a barcoding step was performed using the Native Barcoding Expansion 1-96 (EXP-NBD196) kit (ONT, UK). Library preparation was performed using the native barcoding genomic DNA with the ligation sequencing kit (SQK-LSK109) according to the SQK-LSK109 protocol (ONT, UK). Libraries were sequenced on a GridION (ONT, UK) device using R9.4.1 flow cells. Two independent sequencing runs were performed with 7 and 10 isolates per flow cell.

### Multilocus phylogenetic analysis

To confirm the species of our isolates, a phylogenetic analysis was conducted. We identified and extracted four essential housekeeping genes (*atpA*, *recA*, *rpoA*, and *rpoD*) from both the type strains and the 17 isolates by utilizing the Basic Local Alignment Search Tool (BLAST) accessible through the National Center for Biotechnology Information (NCBI). Subsequently, these genes were used to conduct a multilocus phylogenetic analysis (MLPA). We aligned the concatenated gene sequences using ClustalW, and the phylogenetic tree was constructed using the neighbor-joining method with default parameters and 1,000 bootstrap replications in MEGA v.7 (26).

### Genome assembly and annotation

The base-calling of fast5 files was performed using Super Accuracy mode in Guppy, an ONT software. Quality and read length were evaluated using pycoQC v2.5.2 (27) and Nanoplot v1.41.0 (28). The reads were error-corrected using NECAT v0.0.1 correction step (29) with default parameters. Corrected reads were assembled, and the resulting assemblies were circularized using NGSEP Assembler v4.3.1 with default parameters (30). The genes used for circularization were *rpoB* for chromosome I and *oriC* for chromosome II. Assembly quality statistics information and completeness were assessed using QUAST v.5.0.2 with default parameters (31) and BUSCO v.5.3.2 (32), setting the lineage to -l vibrionales_odb10.

The genome-wide average nucleotide identity (ANI) was calculated for the 17 isolates of *Vibrio* using FastANI with default parameters (33). Complete genomes of the type species were used as references (see Table S4), along with the complete genomes of the isolates (see Table S2). The output was converted to a symmetrical matrix and plotted in a heatmap with hierarchical clustering in R v 3.6.1 (R Core Team, 2019) using the packages reshape2, ComplexHeatmap (34), and gplots.

Genomes belonging to the same species were aligned using NGSEP GenomesAligner v4.3.2 with default parameters (35). The annotation was performed using the PATRIC annotation tool v 3.6.2 (36), and genes were grouped into gene families using in-house scripts, selecting genes related to T3SS, T6SS, exotoxins, and antibiotic resistance for further analysis. Additionally, we employed the virulence factor database (VFDB) (37) to confirm the presence of genes associated with T3SS, T6SS, and exotoxins.

Furthermore, we conducted a targeted investigation focused on specific genes of interest. We used BLAST, accessible through the NCBI, to examine effector genes associated with both T3SS1 (*vopQ*, *vopS*, *vopR*, and *VPA0405*) and T3SS2 (*vopC*, *vopT*, *vopL*, *and vopV*). Additionally, we systematically searched for genes encoding proteins related to T6SS, including T6SS1, T6SS2, T6SS3, and T6SS4 (see Table S5). Furthermore, we utilized the public database SecRet6 v.3 with default parameters (38) to validate the presence, location, and specific type of T6SS.

The results were converted to a matrix and plotted to a heatmap using R version 3.6.1 (R Core Team, 2019) with the packages reshape2, ComplexHeatmap (34), RColorBrewer, pheatmap, and gplots.

## RESULTS

### General features of the *Vibrio* genomes associated with *A. tuberculosa*

A total of 69 isolates were obtained by plating the 14 *A. tuberculosa* samples obtained from two sampling sites on the Colombian Pacific coast. Based on the preliminary identification of the isolates at the genus level, performed by analysis of 16S rRNA sequences, of approximately 1,263 bp, using the BLASTn algorithm (39), we selected 17 isolates identified as *Vibrio* spp. (9 from Buenaventura and 8 from Iscuandé). The *Vibrio* sp. was the second most prevalent genus after *Pseudoalteromonas* (26 isolates) among our collected isolates, even though the medium used was not selective for *Vibrio*. Additionally, we identified other genera with fewer than 10 isolates each, including *Acinetobacter*, *Bacillus*, *Beneckea*, *Enterobacter*, *Photobacterium*, *Pseudomonas*, *Shewanella*, and *Tenacibaculum*. For this study, we selected *Vibrio* isolates based on their potential as a pathogenic group.

A phylogenetic study of the 16S rRNA sequences showed that our isolates corresponded to two main clades, i.e., Harveyi clade (*n* = 16) and Fluvialis clade (*n* = 1) (Fig. S1). Further analysis was also conducted using genome sequencing to identify all isolates at the species level.

The 17 *Vibrio* genomes were assembled into two to three contigs each, with an average total length of 5.25 Mbp (average N50 of 3.35 Mbp, %GC of 45.42), and 4,955 genes annotated on average, as expected. Only two isolates, I24A and I15A, possessed three contigs in their assembly, with the third contig representing a plasmid. Complete statistics of the assemblies generated by QUAST v.5.0.2, such as the number of contigs, total length, N50, %GC, and the number of conserved genes according to BUSCO v.5.3.2, are reported in Table S6. For each assembly, the largest contig (~3.2 Mbp) containing the *rpoB* gene was assigned to chromosome I, and the following contig (~1.8 Mbp) containing the *oriC* gene was assigned to chromosome II.

### Species identification using FastANI and multilocus phylogenetic analysis

**Fig 1:**
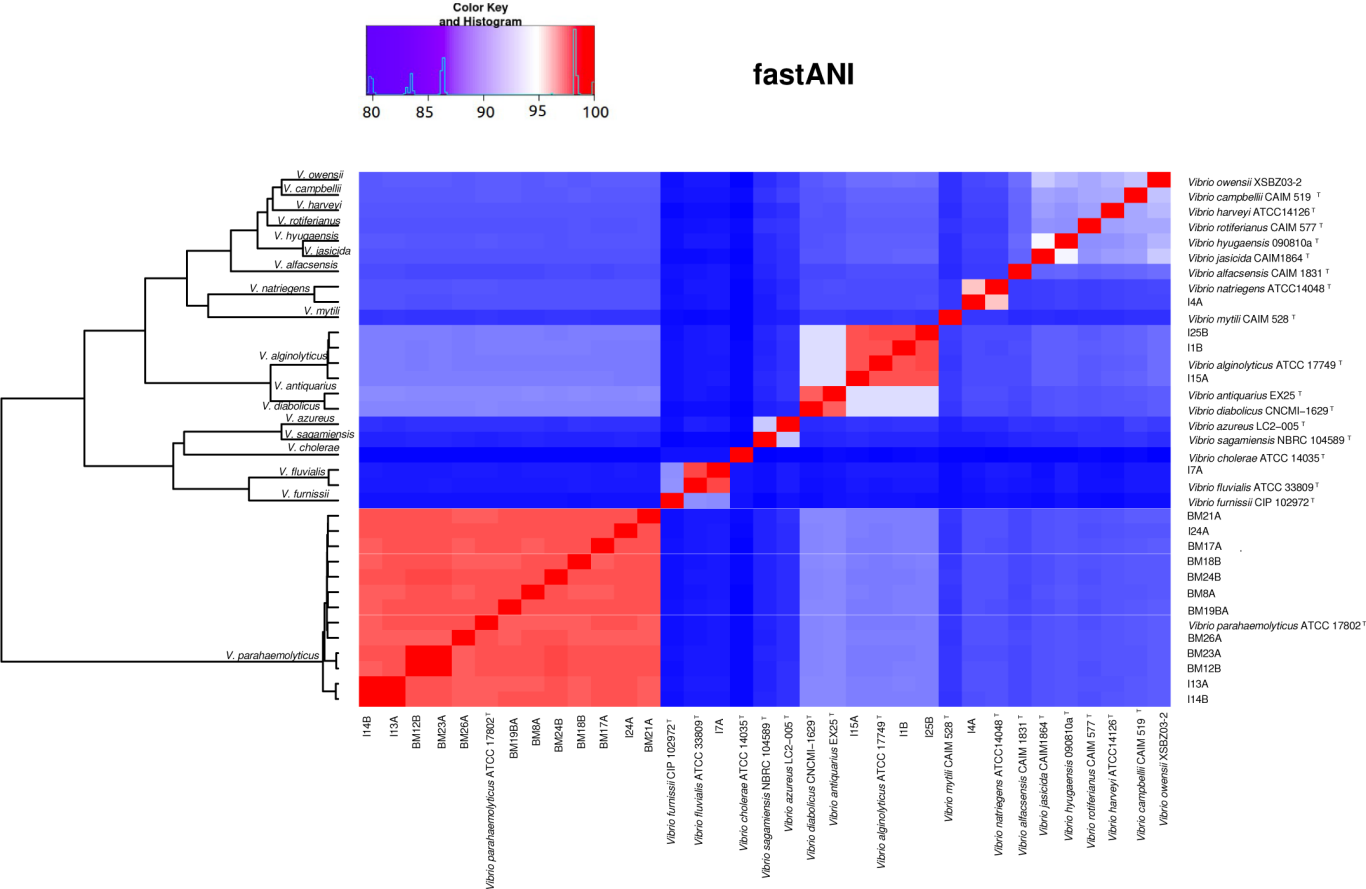
Hierarchical clustering and heatmap based on the whole-genome ANI calculated for the genomes of 17 *Vibrio* spp. isolates of the study and publicly available genomes. Due to the unavailability of a fully sequenced *Vibrio owensi* CAIM 1854 type strain genome, we used the reference genome *V. owensii* XSB Z03 for this analysis. ^T^, Type strain. Name of the isolates begins with I or BM (I, Iscuandé; BM, Buenaventura). Value: Displays values of similarity (value).

**Fig 2:**
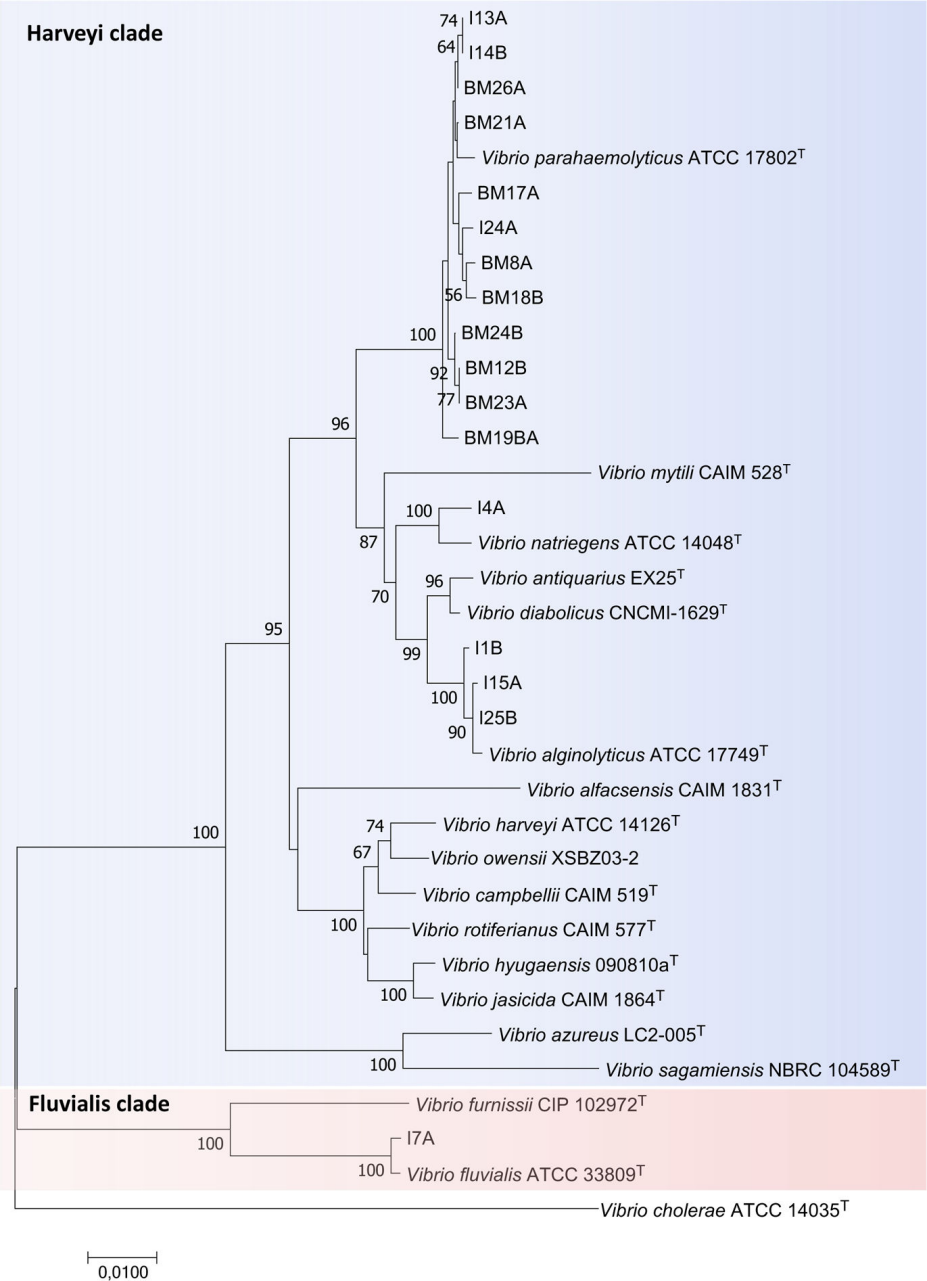
Multilocus phylogenetic analysis conducted using concatenated sequences of four housekeeping genes (3,495 bp) from *Vibrio* genomes (*atpA*, *recA*, *rpoA*, and *rpoD*). The phylogenetic tree was constructed using the neighbor-joining method. Numbers at nodes indicate bootstrap values (percentage of 1,000 replicates). Bar 0.01 estimated nucleotide substitutions per site. Due to the unavailability of a fully sequenced *Vibrio owensi* CAIM 1854 type strain genome, we used the reference genome *V. owensii* XSB Z03 for this analysis. The Harveyi clade is highlighted in blue, and the Fluvialis clade in red. ^T^, Type strain.

To determine species-level classifications, we utilized average nucleotide identity based on complete genome sequences (Fig. 1). This assignment was further confirmed through MLPA with concatenated sequences of four housekeeping genes (*atpA*, *recA*, *rpoC*, and *rpoD*) (Fig. 2), extracted from our isolates and the genomes of *Vibrio* type strains. The results from both ANI and MLPA indicated that our isolates belonged to the following species: *V. parahaemolyticus* (*n* = 12), *V. alginolyticus* (*n* = 3), *V. fluvialis* (*n* = 1), and *Vibrio natriegens* (*n* = 1).

### Presence of antibiotic resistance genes in *Vibrio* environmental isolates

**Fig 3:**
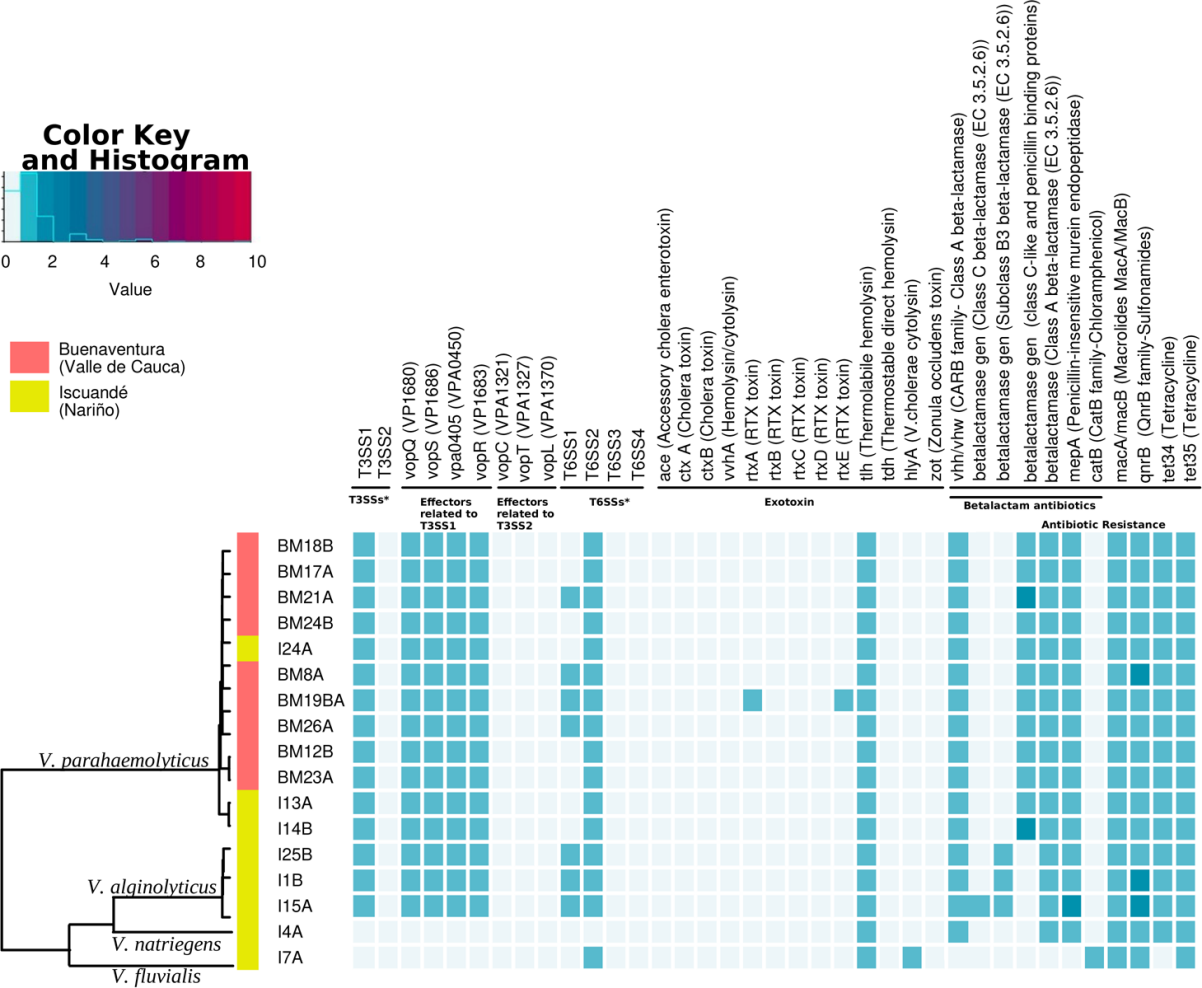
Representation of the genes related to T3SS, T6SS, exotoxins, and antibiotic resistance in the analyzed isolates. Each column represents a specific gene or gene family. *See Fig. S2 for more details on T3SS and T6SSs. Color key: Represents the number of times it was found in the genome (value).

We found genes that confer resistance to beta-lactam antibiotics, such as penicillins, cephalosporins, monobactams, and carbapenems. Except for *V. fluvialis*, all isolates carried genes belonging to at least one class of beta-lactam resistance genes. Following Ambler classification, enzymes belonging to classes A, C, and D employ serine in the process of β-lactam hydrolysis, whereas class B metalloenzymes rely on divalent zinc ions for substrate hydrolysis (40). We observed the presence of resistance genes of class A, class B, and class C, but no evidence of class D genes. All isolates of *V. parahaemolyticus*, *V. alginolyticus*, and *V. natriegens* harbored class A beta-lactam resistance genes. Furthermore, we identified genes related to subclass B3 beta-lactamase of class B in isolates of *V. alginolyticus*. Moreover, class C beta-lactamases were detected in only one of the *V. alginolyticus* isolates (I15A) (Fig. 3).

All isolates contained the *tet35* gene, whereas the *tet34* gene was present in all isolates except for *V. fluvialis*; these genes contribute to tetracycline resistance (41). We also found a *catB* family gene exclusively in *V. fluvialis*, for resistance to chloramphenicol, and the *qnrB* family genes involved in resistance to fluoroquinolone antibiotics in all isolates, except for *V. natriegens* (Fig. 2). These *qnrB* genes encode pentapeptide repeat proteins that protect the quinolone targets, indicating a potential mechanism for resistance to fluoroquinolone antibiotics in these strains.

Other types of resistance determinants found in all isolates included the *macA* and *macB* genes associated with specific macrolide efflux proteins. MacA is an efflux pump that provides resistance to macrolide antibiotics, and MacB is an ATP-binding/permease protein involved in exporting macrolides (42) (Fig. 3).

### The landscape of Type III (T3SS) and Type VI (T6SS) Secretion Systems in *Vibrio* isolates

We also looked for the presence of genes for T3SS and T6SS Secretion Systems that are critical for various cellular processes, including host interactions and bacterial competition, such as the acquisition of essential nutrients, biofilm formation, and defense against competing microorganisms. All our isolates, except for the *V. fluvialis* and *V. natriegens* isolates, contained structural genes associated with the T3SS (Fig. 3). The absence of a T3SS in *V. fluvialis* and *V. natriegens* aligns with recent findings (43). However, we found only one set of genes, despite previous reports of two T3SS variants (T3SS1 and T3SS2) in certain strains of *V. parahaemolyticus* and *V. alginolyticus* (44).

To further characterize the T3SS1 in our isolates, we looked for effector proteins using SecRet6 v.3 and BLASTn. The analysis revealed the presence of genes *vopQ*, *vopR*, *vopS*, and *vpa0405* in all isolates of *V. parahaemolyticus* and *V. alginolyticus*. T3SS1 was consistently located on chromosome I. As expected, the *V. fluvialis* and *V. natriegens* genomes did not yield any hits, in line with our findings indicating the absence of T3SSs in these isolates. Furthermore, none of the four effectors associated with T3SS2 (*vopC*, *vopT*, *vopV*, and *vopL*) were detected in any of the isolates, suggesting the absence of T3SS2 (Fig. 3).

Variable results were also found for T6SS, a contact-dependent protein secretion system utilized by Gram-negative bacteria to deliver toxic effector proteins to neighboring bacterial or host cells, which plays a significant role in interbacterial competition and interactions with eukaryotic cells (13). The isolate of *V. fluvialis* and 8 of 12 *V. parahaemolyticus* isolates possess only T6SS2, whereas the other four isolates (BM21A, BM8A, BM19BA, and BM26A) possess both T6SS1 and T6SS2, like all the three isolates of *V. alginolyticus*. This was confirmed by SecRet6 v.3 and the presence of characteristic genes *vipA1* for T6SS1 and *vipA2* for T6SS2 (45, 46). All isolates with T6SS2 showed the presence of *vasH* and *vasL* genes (Fig. S2).

In *V. parahaemolyticus*, T6SS2 was located on chromosome II in all isolates, whereas those possessing T6SS1 also had it on chromosome I. In all *V. alginolyticus* isolates, both secretion systems (T6SS1 and T6SS2) were located on chromosome I, and in the *V. fluvialis* isolates, T6SS2 was located on chromosome II. These findings were further validated using the public database SecRet6 v.3 (38), which allowed us to determine the chromosomal locations and confirm the specific T6SS variants present. Additionally, it allowed us to observe the positioning and localization of the structural genes of T6SS in each isolate and the reference strains. We can see that the gene location and orientation within the T6SS1 and T6SS2 gene clusters are highly consistent (Fig. S3). The absence of genes related to T6SSs in the *V. natriegens* isolate strongly indicates the absence of a functional T6SS (Fig. 3). No other types of T6SSs, such as T6SS3 or T6SS4, that have been reported in other *Vibrio* species (47 ), were found in any isolates.

### Presence of virulence-associated exotoxins in non-cholera *Vibrio* isolates

We also investigated the presence of exotoxins that could enhance the virulence of *Vibrio* species. Within the well-known hemolysins, we found that all isolates had the gene for thermolabile hemolysin (*tlh*) (48), whereas the hemolysin gene *hlyA* (49) was exclusively identified in the *V. fluvialis* isolate. Only one *V. parahaemolyticus* isolate (BM19BA) was found to harbor genes associated with the RTX toxins, a group of toxins recognized for their cytotoxic effects (50). Specifically, we detected the genes *rtxA*, which encodes a toxin molecule, and *rtxE*, which encodes a structural protein (51–53) (Fig. 3).

Furthermore, we did not find the genes for accessory cholera enterotoxin (*ace*), cholera toxin (*ctxA/B*), hemolysin/cytolysin (*vvhA*), and zonula occludens toxin (*zot*) in our isolates. These genes encode other exotoxins and are typically reported as virulence factors (54, 55).

These findings highlight a diverse range of exotoxins among the studied *Vibrio* isolates, potentially contributing to their virulence and pathogenicity. Further research is needed to fully understand the functional role of these exotoxins and their impact on *Vibrio* infections.

## DISCUSSION

This study represents the first report of sequenced genomes of *Vibrio* isolates obtained from *A. tuberculosa*. In this study, 17 *Vibrio* isolates were subjected to genome sequencing, and of these, 16 were classified within the Harveyi clade. Among the Harveyi clade isolates, 75% were identified as *V. parahaemolyticus* (*n* = 12), with *V. alginolyticus* (*n* = 3) and *V. natriegens* (*n* = 1) being less prevalent. Only one isolate, *V. fluvialis*, belonged to the Fluvialis clade, making it the study’s only representative of this clade. These results were obtained through the MLPA and FastANI analyses (Fig. 1 and 2).

The Harveyi clade encompasses both pathogenic and nonpathogenic bacteria. Among our isolates, we identified species that have been reported as pathogens in both humans and aquatic organisms, such as *V. alginolyticus* and *V. parahaemolyticus. V. parahaemolyticus*, a prominent cause of seafood-associated bacterial gastroenteritis worldwide (56), has also been documented as a pathogen in shrimp (57–59) and has been associated with significant mortality in mollusks (60, 61). *V. alginolyticus* has been reported to cause superficial wound infections (62) and is linked to mass mortality events in mollusks (61, 63, 64). However, it should be noted that these species, which could also be nonpathogenic, are commonly found in water sources and as part of the microbiota of aquatic animals (65, 66).

Among our isolates, *V. natriegens* is the only species that has not been reported as a pathogen so far. It is recognized for its exceptional growth rate and potential use as an alternative chassis organism to *Escherichia coli* for molecular cloning and protein expression (67–69).

As the sole representative of the Fluvialis clade, we identified one isolate of *V. fluvialis*. This species has been reported as a human pathogen and an emerging foodborne pathogen, although it is found more commonly as a nonpathogenic bacterium associated with marine environments (70, 71).

To obtain insight into the genetic factors contributing to *Vibrio* bacterial virulence and survival strategies in their specific ecological niche, we annotated genes involved in antimicrobial resistance, T3SS, T6SS, and coding for exotoxins.

Beta-lactams have been one of the most prescribed antibiotics in the world, which is one of the possible reasons bacteria have developed resistance mechanisms (72). In our study, 94% of the isolates had some or several resistance mechanisms, either serine-β-lactamases or metallo-β-lactamases, which hydrolyze the β-lactam ring, conferring resistance to antibiotics such as penicillins, cephalosporins, carbapenems, and monobactams. Previous reports have also identified β-lactamases among the four most prevalent antibiotic resistances in *Vibrio* spp., along with resistance to tetracycline, sulfonamide, and streptomycin (73). However, we did not find genes associated with sulfonamide resistance in the genomes analyzed in this study.

Additionally, we detected the gene associated with chloramphenicol CatB-family (*catB*), chloramphenicol O-acetyltransferase, exclusively in *V. fluvialis*. The reported number of chloramphenicol-resistant isolates in *Vibrio*, as reported in other studies, typically ranged from 1% to 10%, consistent with our findings (73, 74) (Fig. 3).

Several studies have reported a significant incidence and prevalence of quinolone and tetracycline resistance genes among isolates of *Vibrio* species obtained from environmental samples (73). In our case, the tetracycline resistance gene *tet35* was detected in all isolates, and *tet34* was found in most (16 out of 17) of the isolates, indicating a widespread occurrence of this resistance mechanism. The *qnrB* family genes, which can confer resistance to fluoroquinolones, were identified in all isolates except in *V. natriegens*. The presence of the genes that encode resistance to tetracycline and quinolone was not unexpected, as many bacterial strains are known to be widely resistant to this antibiotic (41, 73). This resistance may be attributed to the fact that fluoroquinolones, like tetracyclines, take a considerably longer time to degrade in the environment (75). These findings underscore the importance of closely monitoring the prevalence of antibiotic resistance genes in environmental bacteria, especially since quinolones, along with many β-lactams, sulfonamides, and tetracyclines, are classified as critically important for treating human diseases due to their significance in combating bacterial infections (76).

The discovery of these antimicrobial resistance genes in our *Vibrio* isolates has significant implications for human health and the ecology of marine ecosystems, as previously reported in studies. These results suggest that environmental *Vibrio* species serve as reservoirs for antimicrobial resistance genes. From reservoirs, these determinants could spread to other bacteria in the marine environment through horizontal gene transfer. However, it is crucial to emphasize that the mere presence of these genes does not inherently assure effective bacterial resistance to antibiotics. To substantiate these phenotypes, it is imperative to conduct laboratory-based resistance assays, as prior research has revealed that, despite the genetic predisposition for resistance, bacteria may still exhibit susceptibility using *in vitro* assessments (77). Thus, it is of utmost importance to carry out testing to ascertain the resistant phenotype of these bacteria. These results also underscore the need to investigate diverse alternatives to antibiotic usage, including bacteriophages, to effectively mitigate resistance and safeguard the efficacy of clinical treatments and aquaculture practices.

In addition to antimicrobial resistance determinants, we identified genes associated with T3SS and T6SS Secretion Systems in *Vibrio* species. The T3SS is a highly conserved complex found on the surface of Gram-negative bacteria (78). In *Vibrio* species, T3SS is a crucial virulence factor, allowing the bacteria to inject effector proteins directly into host cells, facilitating colonization and evasion of the host immune response. This system plays a pivotal role in pathogenicity and the establishment of infections in both aquatic organisms and humans (79). The T6SS was initially described as a bacterial virulence determinant (80), and results have shown that many T6SSs are used as an antibacterial determinant in interbacterial competition (81, 82).

T3SS was found only in *V. parahaemolyticus* and *V. alginolyticus* isolates, which exhibited a singular instance of T3SS. Contrary to prior investigations, we did not find two T3SSs. These studies have indicated the coexistence of two distinct T3SSs within certain strains, specifically T3SS1 and T3SS2 harbored on separate chromosomes (83). In our case, all isolates of both *V. parahaemolyticus* and *V. alginolyticus* presented only T3SS1 located at chromosome I, whereas T3SS2 was notably absent (Fig. S2). This result was confirmed by the identification of the genes for effectors associated with T3SS1 (*vopQ*, *vopS*, *vpa0450*, *and vopR*) and the absence of those involved in T3SS2 (*vopC*, *vopT*, *vopL*, and *vopV*). Finally, the presence of T3SS in all our *V. parahaemolyticus* strains is consistent with previous work showing conservation of T3SS1 across all environmental and clinical *V. parahaemolyticus* strains, irrespective of their pathogenic potential (Fig. 3) (80, 84).

All *V. parahaemolyticus*, *V. alginolyticus*, and *V. fluvialis* isolates analyzed were found to possess T6SS2, but only seven of these contained both T6SS1 and T6SS2. T6SS1 was only identified in 4 out of 12 *V. parahaemolyticus* isolates (BM12A, BM8A, BM19BA, and BM26A) and in all three *V. alginolyticus* isolates (I25B, I1B, and I15A). The T6SS1, reported in most *V. parahaemolyticus* strains, mediates antibacterial toxicity during interbacterial competition by delivering effectors (81, 82). This variant is present in most clinical isolates but less common in environmental ones (85, 86). With respect to the T6SS2 system in *V. parahaemolyticus*, studies have revealed that it is universally present in all isolates, with the co-occurrence of T6SS1 and T6SS2 being more common in clinical isolates (86). A comprehensive analysis of over 1,700 *V*. *parahaemolyticus* genomes reported that approximately 99% of these genomes contained T6SS2 and around 70% featured T6SS1. Additionally, two additional clusters, T6SS3 and T6SS4, were identified, flanked by DNA mobility genes, suggesting potential acquisition through horizontal gene transfer. Notably, the distribution of these two latter clusters was quite limited, with a prevalence of less than 3%. Consistent with this limited distribution, none of our isolates contained these clusters (T6SS3 and T6SS4), even though they all consistently exhibit T6SS2 and, albeit in a lower proportion, T6SS1. Structurally, our isolates exhibit high similarity in terms of gene location and orientation in the T6SS1 and T6SS2 gene clusters when compared across species and with the reference species (Fig. S3). Notably, *V. natriegens* lacked both secretion systems, T3SS and T6SS, consistent with reports indicating its nonpathogenic nature (Fig. 3) (87).

Finally, we also identified genes encoding exotoxins, although their presence varied across genomes. All isolates possessed the *tlh* gene, encoding a thermolabile hemolysin (TLH) ubiquitous among Vibrionaceae species. This hemolysin is known to cause lysis of red blood cells (17, 48) and is found in clinical and environmental strains of *V. parahaemolyticus* (48). However, other hemolysins such as thermostable direct hemolysin (*tdh*) and trh-related hemolysin (*trh*), associated with the hemolytic and cytotoxic activities of *V. parahaemolyticus* in the host (17, 88), were not found in any of the isolates (Fig. 3).

In addition to these toxins, one isolate of *V. parahaemolyticus* (BM19BA) also carried *rtxA* and *rtxE* genes for RTX toxins. RTX toxins, commonly associated with the Type I Secretion System (T1SS) in Gram-negative bacteria, have various effects like the formation of pores, hemolysis, and cytotoxic activity (51–53, 89). They contribute to the virulence of different *Vibrio* species, including *V. cholerae* and *V. vulnificus* (51, 89). Previous studies have reported the presence of RTX toxins in *V. vulnificus*, which have been shown to induce acute cytotoxicity upon contact with host cells (51). RtxA is a toxin and a crucial virulence factor, which has been reported in *V. cholerae* (52). RtxE has been characterized as a transport ATPase, an essential component of the T1SS, contributing to virulence *in vitro* and in murine models for *V. vulnificus.* RtxA is secreted by rtxE, and the disruption of *rtxE* inhibits the secretion of RtxA to the cell exterior, leading to a significant reduction in cytotoxic activity against epithelial cells *in vitro* (53). The gene encoding HlyA (α-hemolysin), the most well-characterized RTX hemolysin protein and an important virulence factor, was found exclusively in the *V. fluvialis* isolate. HlyA functions as both a cytotoxin and an enterotoxin (Fig. 3) (49). Overall, these results suggest that there are differences in determinants among the isolated strains that could impact their potential virulence determinants and antibiotic resistance genes.

These findings emphasize the potential risks associated with the persistence and dissemination of bacteria that could threaten both human and animal health. Consequently, we strongly recommend the implementation of strategies aimed at preventing disease outbreaks while protecting the sustainable use of these mangrove ecosystems. The risks associated with virulent strains of *Vibrio* spp. can be effectively mitigated by prioritizing practices such as cooking bivalves, avoiding the consumption of raw animals, and regular monitoring for the presence of bacteria carrying virulence genes. It is essential to take proactive measures to ensure the health of aquaculture species relevant to local populations and the industry’s long-term viability.

## Data Availability

The 16S rRNA genes and genome sequences are available in the open source sequence data repository NCBI under BioProject identification (ID) PRJNA992554.

## SUPPLEMENTAL MATERIAL

Fig. S1 spectrum.02928-23-s0001.pdf 16S rRNA maximum-likelihood phylogenetic reconstruction of the 16S rRNA sequences.

Legends for supplemental figures spectrum.02928-23-s0002.docx Legends for Fig. S1 to S3.

Fig. S2 spectrum.02928-23-s0003.pdf Representation of genes related to the T3SS and T6SS in the analyzed isolates.

Fig. S3 spectrum.02928-23-s0004.pdf Graphic representation of the structural genes within the T6SS.

Legends for supplemental tables spectrum.02928-23-s0005.docx Legends for Tables S1 to S6.

Supplemental tables spectrum.02928-23-s0006.xlsx Tables S1 to S6.

ASM does not own the copyrights to Supplemental Material that may be linked to, or accessed through, an article. The authors have granted ASM a non-exclusive, world-wide license to publish the Supplemental Material files. Please contact the corresponding author directly for reuse.
