# Supplementary material for: Unveiling potential virulence determinants in Vibrio isolates from Anadara tuberculosa through whole genome analyses
Source: Microbiol Spectr. 2024 Jan 8;12(2):e02928-23. doi: 10.1128/spectrum.02928-23 (PMC10846245; doi:10.1128/spectrum.02928-23)
Supplement: Fig. S1 — 16S rRNA maximum-likelihood phylogenetic reconstruction of the 16S rRNA sequences. [file spectrum.02928-23-s0001.pdf]

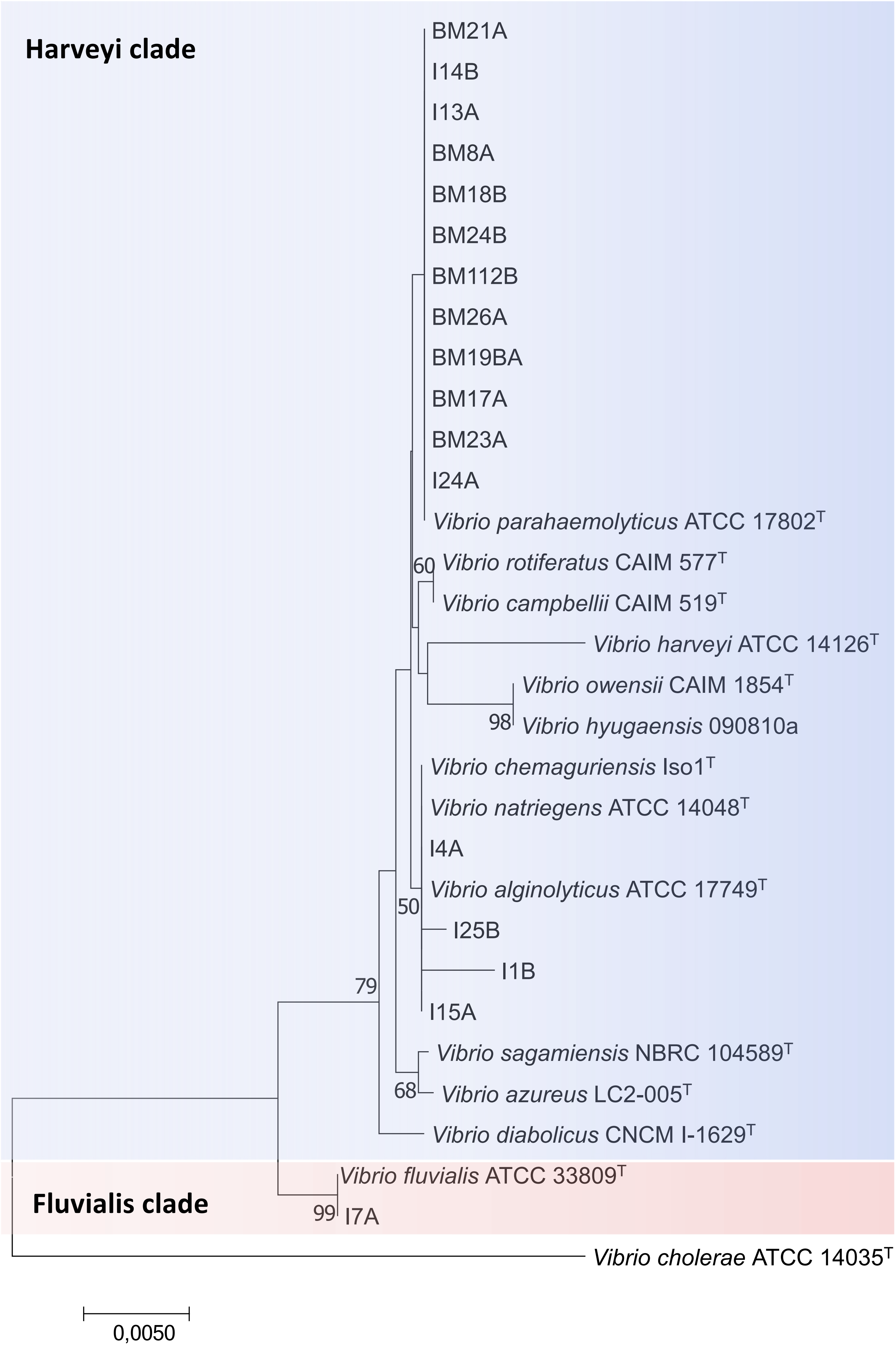

Harveyi clade
BM21A
I14B
I13A
BM8A
BM18B
BM24B
BM112B
BM26A
BM19BA
BM17A
BM23A
I24A
Vibrio parahaemolyticus ATCC 17802T
60
Vibrio rotiferatus CAIM 577T
Vibrio campbellii CAIM 519T
Vibrio harveyi ATCC 14126T
Vibrio owensii CAIM 1854T
98
Vibrio hyugaensis 090810a
Vibrio chemaguriensis Iso1T
Vibrio natriegens ATCC 14048T
I4A
Vibrio alginolyticus ATCC 17749T
50
I25B
I1B
79
I15A
Vibrio sagamiensis NBRC 104589T
68
Vibrio azureus LC2-005T
Vibrio diabolicus CNCM I-1629T
Vibrio fluvialis ATCC 33809T
Fluvialis clade
99
I7A
Vibrio cholerae ATCC 14035T
0,0050
