# Supplementary material for: Unveiling potential virulence determinants in Vibrio isolates from Anadara tuberculosa through whole genome analyses
Source: Microbiol Spectr. 2024 Jan 8;12(2):e02928-23. doi: 10.1128/spectrum.02928-23 (PMC10846245; doi:10.1128/spectrum.02928-23)
Supplement: Legends for supplemental figures — Legends for Fig. S1 to S3. [file spectrum.02928-23-s0002.docx]

**SUPPLEMENTARY FIGURES AND LEGENDS**

**Supplementary Figure 1**. Neighbor-Joining phylogenetic reconstruction of the 16S rRNA sequences, obtained through PCR and sequenced using Sanger technologies, in comparison with *Vibrio* public sequences of the type species. The phylogenetic tree was rooted with V*ibrio cholerae.* The aligned dataset had a length of 1263bp. Bar 0.05 estimated nucleotide substitutions per site. The Harveyi clade is highlighted in blue, and the Fluvialis clade in red.

^T^ Type strains.

**Supplementary Figure 2**. Representation of genes related to the T3SS and T6SS, in the analyzed isolates. Each column represents a specific gene or gene family.

Color Key: Represents the number of times it was found in the genome (Value)

**Supplementary Figure 3:** Graphic representation of the structural genes within the T6SS gene cluster created using SecRet6 v. 3 software. The genome of the type species was utilized to group the isolates for enhanced visualization by species. (T6SS1 or T6SS2)
