# Supplementary figures and images for: Unveiling potential virulence determinants in Vibrio isolates from Anadara tuberculosa through whole genome analyses
Source: Microbiol Spectr. 2024 Jan 8;12(2):e02928-23. doi: 10.1128/spectrum.02928-23 (PMC10846245; doi:10.1128/spectrum.02928-23)

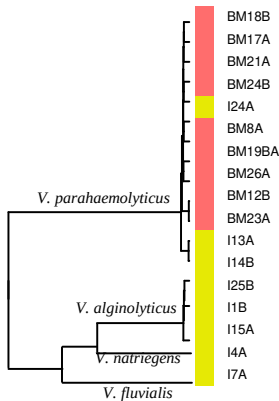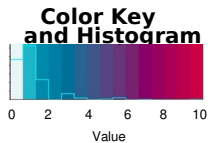

Buena Ventura  
(Valle de Cauca)

Iscuandé  
(Nariño)

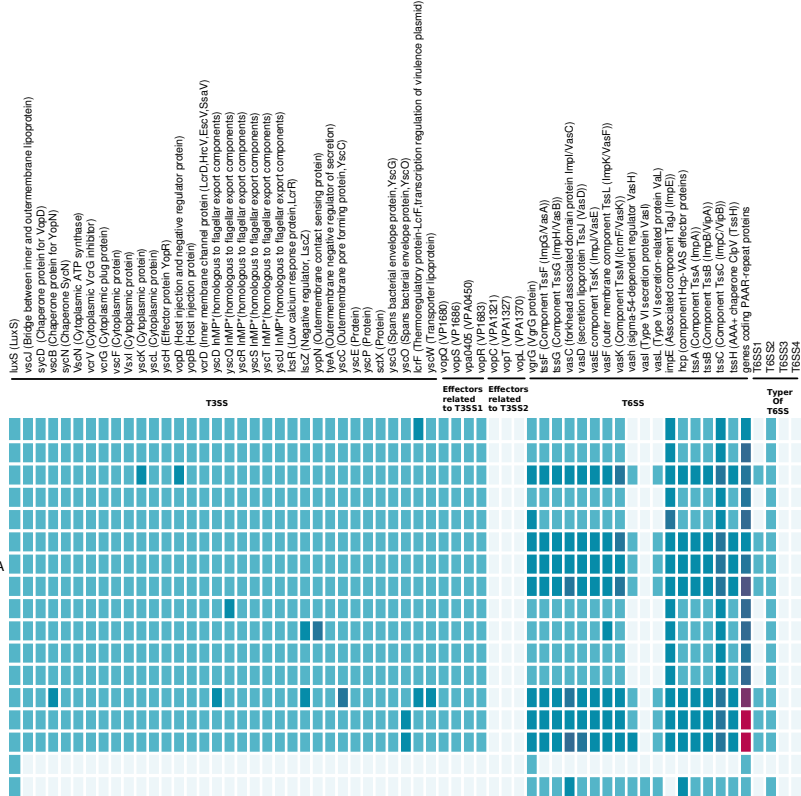

Supplement: Fig. S2 — Representation of genes related to the T3SS and T6SS in the analyzed isolates. [file spectrum.02928-23-s0003.pdf]

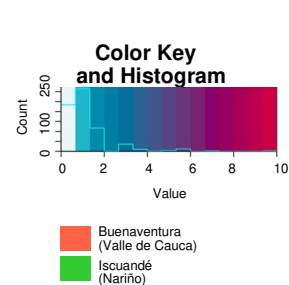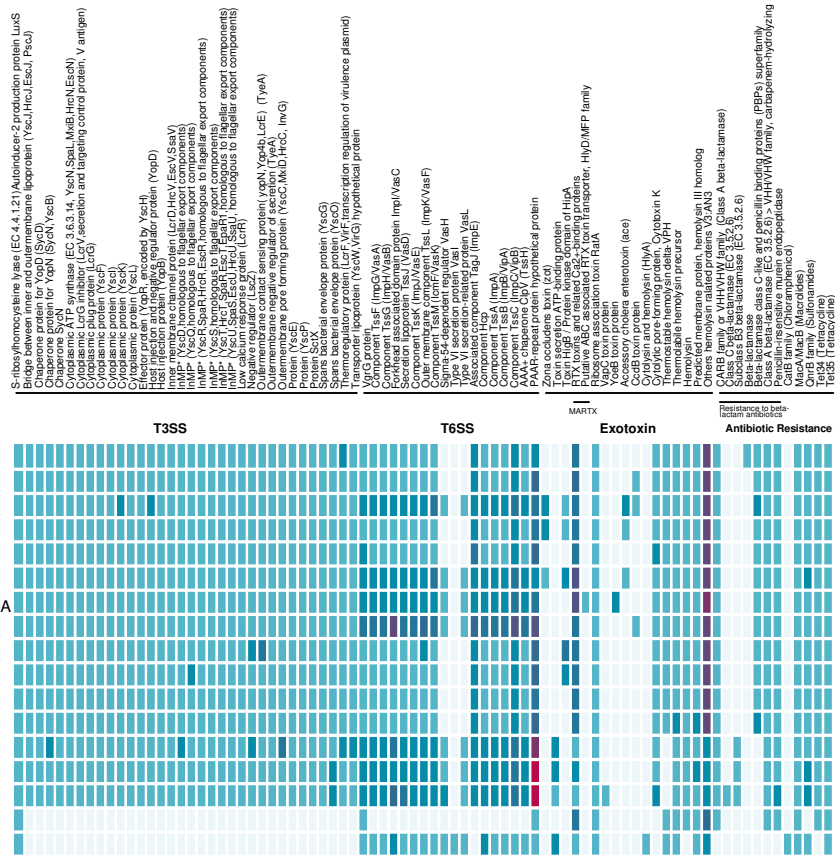

Supplement: Fig. S3 — Graphic representation of the structural genes within the T6SS. [file spectrum.02928-23-s0004.pdf]
