# Supplementary material for: Unveiling potential virulence determinants in Vibrio isolates from Anadara tuberculosa through whole genome analyses
Source: Microbiol Spectr. 2024 Jan 8;12(2):e02928-23. doi: 10.1128/spectrum.02928-23 (PMC10846245; doi:10.1128/spectrum.02928-23)
Supplement: Legends for supplemental tables — Legends for Tables S1 to S6. [file spectrum.02928-23-s0005.docx]

**SUPPLEMENTARY TABLES AND LEGENDS**

**Supplementary Table 1**. *Collection sites. Anadara tuberculosa* specimens collection data

**Supplementary Table 2**. Identification, origin, and GenBank accession numbers of the sequences (16S) and genomes of the *Vibrio* isolates obtained from *Anadara tuberculosa* included in this study.

**Supplementary Table 3.** Accession number of16S rRNA sequences of type strains used to reconstruct the ML tree.

**Supplementary Table 4**. Accession numbers of the complete genomes included in the analysis.

**Supplementary Table 5.** Gen ID used in this study.

**Supplementary Table 6.** Genome assembly features of the genomes generated in this study.
